# Validation study for the Academic Maladjustment Questionnaire on a Romanian sample

**DOI:** 10.3389/fpsyg.2023.1275939

**Published:** 2023-11-15

**Authors:** Ana-Maria Cazan, Maria Magdalena Stan, Aurel Ion Clinciu, Camelia Truţa, Catalin Ioan Maican

**Affiliations:** ^1^Department of Psychology and Education Sciences, Faculty of Psychology and Education Sciences, Transilvania University of Braşov, Braşov, Romania; ^2^Piteşti University Centre, The National University of Science and Technology POLITEHNICA Bucharest, Piteşti, Romania; ^3^Department of Management and Economic Informatics, Faculty of Economic Sciences and Business Administration, Transilvania University of Braşov, Braşov, Romania

**Keywords:** academic maladjustment, dropout intention, academic dishonesty, procrastination, academic performance

## Abstract

**Introduction:**

The problem of academic dropout in the first year of studies represents an important issue for higher education, in that it accounts for an important indicator of quality but also for the negative consequences it produces on individual, institutional and social level. The main aim of the study is to validate and evaluate a robust measure of overall academic maladjustment.

**Method:**

The participants were 809 first-year students from various Romanian universities.

**Results:**

The results showed a reliable version of the instrument with a factorial structure that did not deviate significantly from the authors’ initial model. The exploratory and confirmatory factor analysis revealed a unified score including six dimensions, procrastination, dishonesty – unethical behavior, test anxiety, machiavellian attitude, neuroticism, and somatization. Our results confirmed that besides academic achievement, personal factors are important indicators of adjustment, showing that personal resources management, emotional and behavioral strategies are components of adjustment. Our study revealed a medium and positive correlation between overall maladjustment and academic dropout intention, procrastination seemed to be the most relevant predictor of dropout intention.

**Discussion:**

Academic adjustment acts as a safeguard against dropping out, and it is crucial to acknowledge that most students enter college with the intention of completing their studies.

## Introduction

University represents a vital transition period for youths’ evolution as it includes numerous challenges which pertain not only to academic requirements but also to the change of a new social environment where adaptability is an important factor for academic achievement (Xie et al., 2019). First year students must become active members of university community, through experimenting new roles and social responsibilities and manifesting high levels of autonomy and independence (Credé and Niehorster, 2012). Successful adjustment to academic environment demands has a direct effect upon the stimulation of students’ learning potential and offers direct perspectives upon development in the professional career. Inability to adjust has repercussions not only upon social integration (Xie et al., 2019), but also can determine the phenomenon of dropout.

The problem of academic dropout in the first year of studies represents an important issue for higher education, in that it accounts for an important indicator of quality but also for the negative consequences it produces on individual, institutional and social level. Students who abandon college have few possibilities to develop professional competences which could offer them the chance of a good integration on the labor market. Statistics in USA show that dropout rate for young people between the ages of 16−24 years is of 5.2% in 2021 (National Center for Education Statistics [NCES], 2023), in UE the average of European countries is of 9.6%, with the highest rate being registered in Romania – 15.6% (Eurostat, 2023). Medium and long-term negative consequences reflect in the integration and socio-professional affirmation difficulties (Kaufman et al., 2004) which can generate important socio-economic problems such as high unemployment rate, poor quality in carrying out professional tasks and high costs in social security benefits (Curelaru et al., 2010). Negative effects of dropout rates at the institutional level impact the reduction of financial governmental resources (Herţeliu et al., 2022), and also institutional prestige (Angulo-Ruiz and Pergelova, 2013) since dropout is considered an indicator of a low level of the quality of educational act (Tresman, 2002).

At personal level, dropout can lead to low expectations, negative self-evaluation (Hällsten, 2017), high risks of affecting self-esteem and well-being (Bianchi et al., 2021), the lack of non-cognitive skills (Heckman and Rubinstein, 2001), which associates with a rise in the demotivation of continuing future studies (Delnoij et al., 2021), with a low level of employability (Dunn et al., 2004) and of job performance (Sosu and Pheunpha, 2019).

In the present paper we focus on the main predictor of academic dropout which is academic maladjustment. The topic was previously approached, as we will present below, however, there isn’t a recent integrative framework of academic (mal)adjustment. Baker and Siryk’ s model (1984) and Tinto’s theory (1993) are still the most cited and with large empirical evidence, but recent studies argue that academic adjustment is more contextual and should be analysed as such (Fernández et al., 2017). Therefore, the present study has the following goals: (1) to present a theoretical framework for academic adjustment and its predictors (2) to refine and validate an instrument aimed at measuring academic maladjustment. Our study aims to make several contributions in the field. First, contributing to academic adjustment literature, by exploring the most relevant theories of adjustment to university. Second, we expand academic adjustment concept and measure, on one hand by presenting its negative side, maladjustment and on the other hand adding new dimensions, such as academic dishonesty. Third, we will provide empirical evidence on the relationship between academic adjustment and several variables related to university life, such as academic performance and dropout intention or personal factors such as personality traits. All these contributions are possible by introducing a reliable and valid tool for empirical research on academic maladjustment.

## Literature review

### Models of academic adjustment

Studies in the literature confirm that the first year of study represents an important determinant in the appearance of dropout due to the transition period in the academic adjustment process (Xavier and Meneses, 2022). The transition period is usually associated with an adjustment period (Nicholson, 1990), but also with a high degree of uncertainty (Gurin et al., 2002). Extensive studies identified the factors that contribute to the appearance of the dropout phenomenon, but none of them can be held responsible for the academic dropout. The dropout process is a gradual disconnection process from academic life that starts during the first months and which implies unsatisfactory experience, inability to cope with academic demands, wrong choice of course, and a range of personal factors like financial problems, illness, and family circumstances. Time factors represent the most important barriers correlated to persistence and the main reasons for dropout – time-related conflicts, poor time management skills in ensuring a balance between study and certain social responsibilities (engagement at the workplace and/or in the family life). Much research identifies factors such as low levels of engagement and academic motivation, low levels of academic adjustment, learning difficulties, the lack of learning strategies which lead from absenteeism to dropout at first year students (Truta et al., 2018; Thomas et al., 2021).

Persistence and the effects generated by the university dropout phenomenon, as well as the necessity to improve graduation rates have received increased attention in reference to first year students’ academic adjustment. It is arguable that identifying the causes and predictors of academic maladjustment could determine the development of efficient programs which, through early warning measures will help diminish risk factors in order to prevent academic dropout or absenteeism.

Adjustment refers to the behaviors which allow each individual to deal with the demands and expectations of a new environment, to act and develop new coping strategies (Fernández et al., 2017; Cricchio and Coco, 2022). Academic adjustment is a multifaceted construct which involves the activation of individual skills to meet the demands of the new academic environment and of learning activities (Tanyi, 2002). The new physical, social and personal academic environment involves new rules, demands, expectations and responsibilities. Students must demonstrate that they cope with a competitive learning environment, that through engagement, they can follow educational and institutional goals and can adhere to new social networks. Academic challenges bring students to engage in a more demanding learning activity, to adjust to new teaching and evaluation styles that imply complex cognitive efforts, for which they are not prepared. Furthermore, students must comply with a new type of learning- academic learning which involves the activation of new skills and attitudes- autonomy, responsibility, time management, planning, and organizing learning, etc. At a social level, students must adhere to new social groups, develop new friendships, collaborate with peers and teachers and engage in the new organizational culture. The change in the lifestyle and the development of new routines determine a restructuring of social roles, of personal values with effects on the identity level (Scanlon et al., 2007).

Research showed the fact that the easier the students get through this stage and adjust their expectations to the demands of the new environment, the more likely they engage in study and in academic life. For some students, getting over this process is easy and fast while for others changes are overwhelming and difficult, manifesting academic maladjustment (Cricchio and Coco, 2022). Students’ poor adjustment to the demands of the new environment impacts their psychological and physical health, through manifesting anxiety, depression, high stress levels, low level of self-esteem (Sha et al., 2000; Wintre and Bowers, 2007), eating disorders, alcohol and substance abuse (Ravert, 2009), and also affecting academic performance (Cricchio and Coco, 2022).

Academic adjustment is a process which results in academic achievement (van Rooij et al., 2018). The specialty literature registers numerous models of academic achievement. Tinto (1993) considered that the decision of completing or dropping out of studies is the result of a longitudinal process of simultaneous or successive interactions among family factors, pre-university studies, learning skills and capacities, goals and engagements, institutional experiences, and quality of integration. Positive experiences from academic environment lead to good integration, while negative experiences lead to discouragement, decrease of motivation, academic performance and dropout (Schwartz and Tinto, 1987). Other authors (Pascarella et al., 2004) emphasize the role of psycho-individual variables, considering that learning and cognitive development quality and level are influenced by the quality of the effort the student makes in the learning process. Organizational and structural characteristics of the institution exert an indirect influence over student’s development, their effect being mediated by the institutional environment and social interactions (relation with peers and teachers) (Astin, 1999). In his proposed model, Astin (1999) lays the emphasis on the quality of students’ engagement in the academic achievement process and on the psychological energy students invest in the experience with academic environment. Astin (1999) considered that academic adjustment is another facet of students’ engagement, of the effort they make during the first year of study. Academic achievement is directly influenced by variables like previous academic performance, introversion and agreeableness, emotional stability and openness to experience and indirectly by variables like self-efficacy, locus of control, learning and performance orientation, discipline interest and conscientiousness (McKenzie et al., 2004). Personality variables act directly on academic adjustment through the impact they have upon the choice of studying and of learning strategies. According to the model, academic achievement obtained in the first semester is considered to be a predictor of the results registered in the second semester.

Academic maladjustment approach is less represented in the specialty literature than academic adjustment, the problematics being quite controversial mainly due to its conceptualization in opposition to academic adjustment. Not always low levels of the factors which determine academic adjustment explain the variation academic maladjustment. Academic maladjustment refers to the set of behavioral, social and emotional difficulties which prevent students from adjusting to the new learning environment, from meeting successfully academic demands, from obtaining performance and academic engagement (Jansen and van der Meer, 2012). Students who manifest academic maladjustment report dissatisfaction, disengagement, high level of stress which negatively affects well-being (Cricchio and Coco, 2022), academic achievement (Rienties et al., 2012; Bailey and Phillips, 2016) and academic performance (Baker and Siryk, 1984).

Defining the term academic maladjustment is important because the manner in which it is defined can determine the manner in which it is approached, measured and analyzed (Ashby, 2004). Theories which explain first year students’ academic adjustment (Tinto, 1993; Astin, 1999; McKenzie et al., 2004) are consistent with the idea which emphasize the importance of students’ personal traits reflected in the quality of academic experience and in the decision to complete studies. Students’ academic performance is influenced by the level of academic adjustment and psycho-social factors influence academic adjustment and performance (Sommer and Dumont, 2011). Adjustment to university is not an invariable experience, but a multiplicity of complex experiences in which students’ characteristics interact with the process variables and institutional factors, which must be analyzed in the cultural context within which they are produced (Fernández et al., 2017).

Academic maladjustment is a multifaceted concept, being dynamic and multidimensional, which is explained by the fact that the student enters university with certain personal characteristics (personality traits, motivation, academic prerequisites, and study skills) which are challenged by the interaction with the new environment (academic requirements, peers, teachers, institutional culture) in order to set a fit between their demands, values and expectations and the demands and regulations of university life; consequently, academic maladjustment lead to academic failure (Astin, 1999; Pascarella et al., 2004; van Rooij et al., 2018).

As previously mentioned, the literature predominantly emphasizes academic adjustment, with relatively fewer studies focusing on academic maladjustment. Its relevance in understanding and approaching the dropout phenomenon is undoubted, as it leads to a series of negative consequences both at academic and emotional levels. For example, in a previous study academic dishonesty was found to be consistently associated with low levels of academic adjustment (Clinciu et al., 2021), but there is still a need for research to clarify if this behavior is a dimension of academic maladjustment, a factor that contributes to it or a possible consequence of it. The understanding and conceptualization of academic maladjustments should start from an overview of the predictors for which the literature offers consistent empirical support.

Theoretical models in the field of academic adjustment and achievement affirm that academic adjustment predictors fall into three categories: academic, social/environmental and personality factors. Academic factors include a series of variables such as aptitude and ability, study skills and test anxiety, academic motivation, self-efficacy and attribution, social/environmental factors refer to variables such as social support, life stress, campus environment, work involvement, family variables, and academic environment, while personality factors include personality characteristics, self- esteem, locus of control and trait anxiety (Fong et al., 2017).

### Predictors of academic (mal)adjustment

#### Demographic characteristics

Many studies in the specialty literature analyzed background characteristics in predicting adjustment and academic achievement (Tinto, 1993; Wintre and Yaffe, 2000). The effect of predictors such as gender, socio-economic status, age, marital status, first generation university students over academic adjustment was identified as being mediated by social support (Hertel, 2002).

#### Academic prerequisites

Studies showed the essential role of academic adjustment in the prediction of academic performance and of dropout occurrence. Pupils who had good results in high school or at standardized tests adapt easier to the requirements of the new academic environment. Scores at Scholastic Assessment Test (SAT) and American College Testing (ACT) reflect many general cognitive skills such as the capacity to process new information and to acquire new skills. High grades in high school reflect solid cognitive skills which facilitate academic adjustment. Persistence and academic achievement had been most frequently predicted by cognitive variables, respectively, grade point average (GPA) and SAT scores. The grades from high-school and scores at ACT and SAT maintained a more influential predictive relation than study skills, motivation and personality characteristics (Clinciu, 2019).

Academic adjustment represents a mediating variable upon academic performance (van Rooij et al., 2018). Academic adjustment explained the variation of results beyond secondary school GPA (McKenzie et al., 2004). Recent research (van Rooij et al., 2018) detected the presence of a significant variation in academic adjustment which is not explained by the scores at the tests. A student’s GPA reflects how well he performs in cognitive tasks, while the number of European Credit Transfer and Accumulation System (ECTS) reflect the number of credit points students obtain upon completing an academic discipline; hence students with a high GPA and ECTS score can abandon while students with a low GPA and with a low number of ECTS credit points can choose to continue their studies.

#### Personality traits

A variety of individual traits can facilitate or hinder the process of academic adjustment (Wintre and Sugar, 2000; Credé and Niehorster, 2012). The term of academic adjustment includes social and personal aspects related to students’ academic experience: readiness to meet the academic demands, a clear sense of purpose and general satisfaction with the academic environment (Baker and Siryk, 1984).

The relation between personality factors and academic adjustment is not well represented in the literature. The role of personality factors can be better represented through the concept of academic achievement. Among personality traits, conscientiousness has a consistently positive association with academic achievement (Paunonen and Ashton, 2001; Chamorro-Premuzic and Furnham, 2003; Poropat, 2009; Richardson et al., 2012) and performance in exams (Chamorro-Premuzic and Furnham, 2003). Conscientiousness is associated with goal setting and psychological effort mobilization (Barrick and Mount, 1991), with task-solving, effort regulation and time management (Bidjerano and Dai, 2007), the self-regulating element of conscientiousness is more integral to academic achievement in college (Noftle and Robins, 2007).

Neuroticism relates with poor academic performance (Chamorro-Premuzic and Furnham, 2003), being associated with high levels of anxiety and absenteeism at exams (Richardson et al., 2012). Students with high levels of neuroticism experience emotional states such as anxiety, guilt, uncertainty and self-pity and use denial and withdrawal as coping strategies, manifesting the tendency to cheat in exams and procrastination behaviors (Giluk and Postlethwaite, 2015).

Procrastination, conceptualized as the behavioral tendency to unnecessarily postpone decisions or work tasks (van Eerde, 2003), which refers to a deficient control of impulses, a self-regulation failure (Steel, 2007) represents a negative factor of academic adjustment. Students with high levels of procrastination have the tendency to persist less in academic activities (Richardson et al., 2012).

#### Text anxiety

Test anxiety refers to a set of phenomenological, physiological, and behavioral responses that accompany concern about possible negative consequences or failure on an exam or similar evaluative situation. Students who score high values of test anxiety manifest worry, stress, a tension which leads to a failure in regulation and focus. Test anxiety is associated with negative results of students’ academic achievement (Sommer and Dumont, 2011) and academic performances (Richardson et al., 2012). According to Sommer and Dumont’s studies (2011), text anxiety is associated with low levels of self-esteem, poor study habits, poor study techniques and high procrastination, poor motivation, negative self-evaluation and concentration difficulties. In the studies on academic adjustment, test anxiety was not investigated as a predictor of adjustment, it was only analyzed in connection with academic performance.

#### Dishonesty and unethical behavior

Dishonesty represents unethical academic behaviors such as cheating, plagiarism, or unauthorized help, being a widespread phenomenon in the academic environment (Giluk and Postlethwaite, 2015). Ajzen (1991) elaborates the theory of planned behavior, in which he explains that dishonest academic attitudes mediate between causal agents (demographical variables—i.e., sex, age, psychosocial variables—i.e., religious feelings, self-efficacy, academic variables—i.e., motives to study, faculty of enrollment, strategies for learning and academic achievement, and situational variables—peers’ cheating behavior and peers request for help) and dishonest behaviors considering their effects (Schmelkin et al., 2008; Jurdi et al., 2011). Academic dishonesty is associated with academic maladjustment (Clinciu et al., 2021).

While existing literature has identified these factors as predictors of academic maladjustment, there is still unclear how these predictors interact with each other and how their interactions could influence academic adjustment or achievement. For example, how do personality traits, especially neuroticism, interact with procrastination and with dishonesty? Or are there any differences in these factors due to demographics characteristics? These are some of the research gaps in the literature that warrant further investigations with more stable instruments that cover all relevant dimensions of academic adjustment.

### Measuring adjustment to university

The concern for the manner in which students tackle the challenges of the new academic environment with the aim to improve their experience during the first year of study is reflected in the modality of evaluating academic adjustment. The problematics of academic adjustment conceptualization generated an array of modalities and techniques of measuring the construct. Some researchers focused on a single element to measure the whole construct, whereas others focused upon certain facets of the construct. Table 1 shows a synthetic presentation of the main measuring instruments of academic adjustments available in the specialty literature.

**TABLE 1:** Instruments measuring academic adjustment.

Instrument (no. of items, authors)	Sample	Conceptualization/Dimensions	Criterion validity with:
Adjustment to College (52 items) Revised as: ***Student Adaptation to College Questionnaire*** (SACQ), (Baker and Siryk, 1984) (67 items)	Three freshman classes from 3 successive academic years Freshmen	Adjustment = Coping responses to the demands of the college experience. Adjustment is multifaceted with three dimensions: *(a) academic adjustment* – attitudes toward academic goals and the academic work; how well students are applying themselves to their academic work; the effectiveness or sufficiency of their academic efforts; and the acceptability to them of their academic environment and what it is offering them (b) *social adjustment* – the extent and success of social activities and functioning in very general terms; interpersonal relationships; social relocation; acceptability of the social environment to the student (c) *personal-emotional adjustment* – asks students how they are feeling both psychologically and physically (d) *institutional adjustment* – demands of the transition experience in a general way; institutional and/or goal commitment	Attrition = non-enrollment of the beginning of the third semester for any reason. Appeals for psychological services Freshman year grade point average Election to an academic honorary society Application for dormitory positions
***College Adjustment Rating Scale*** (CARS), (Zitzow, 1984) (100 items)	College students	Adjustment is conceptualized as the level of stress within academic, social, personal, and family-home environments. The scale measures the occurrence of stressful events in students’ lives and the self-perceived intensity of the stress. Four domains or environments which particularly relevant for college students: (a) *academic*, (b) *social*, (c) *personal*, (d) *family-home*.	
***Advanced Students’ Adaptation to College*,** (Van Rooijen, 1986) (18 items)	Students in second, fourth and sixth year of study	Unidimensional	Psychosomatic stress Depressive mood Satisfaction with various areas of well-being Drinking problems Life satisfaction Loneliness Interpersonal helplessness Establishing interpersonal relations Social risk-taking Approval-seeking
***College Adjustment*** ***Test*** (CAT), (Pennebaker et al., 1990) (19 items)	Freshmen	The adjustment was conceptualized as the degree to which students have experienced various thoughts and feelings about coming to college during the week previous to the start of the academic year Three factors: (a) general *negative affect* about coming to college (b) *positive affect* or optimism (c) *home sickness*	
***College Adjustment Scales*** (CAS), (Anton and Reed, 1991) (108 items)		Multidimensional (9 scales) *Anxiety* (AN): A measure of clinical anxiety, focusing on common affective, cognitive, and physiological symptoms. *Depression* (DP): A measure of clinical depression, focusing on common affective, cognitive, and physiological symptoms. *Suicidal Ideation* (SI): A measure of the extent of recent ideation reflecting suicide, including thoughts of suicide, hopelessness, and resignation. *Substance Abuse* (SA): A measure of the extent of disruption in the interpersonal, social, academic, and vocational functioning as a result of substance use and abuse. *Self-esteem Problems* (SE): A measure of global self-esteem which taps negative self-evaluations and dissatisfaction with personal achievement. *Interpersonal Problems* (IP): A measure of the extent of problems in relating to others in the campus environment. *Family Problems* (FP): A measure of difficulties experienced in relationships with family members. *Academic Problems* (AP): A measure of the extent of problems related to academic performance. *Career Problems* (CP): A measure of the extent of problems related to career choice.	
***Academic Adjustment Scale*** (AAS), (Anderson et al., 2016) (9 items)	Higher education students and Student sojourners = individuals who reside abroad to pursue higher education	Adjustment is a multifaceted concept including: (a) *academic lifestyle:* AAS-L – the fit between the individual and their temporary role as a student; (b) *academic achievement:* AAS-A – satisfaction with academic progress and performance, and; (c) *academic motivation:* AAS-M – the drive for the student to continue and complete their academic sojourn	Satisfaction with student’s grade point average (GPA)
***College Adjustment Questionnaire*** (CAQ), (O’Donnell et al., 2018) (22 items)	Higher education students	Multidimensional: The *Educational Functioning* subscale focuses on features of academic functioning, such as performance in classes and achievement. The *Relational Functioning* subscale assesses for adjustment in social aspects of college life and explores social connectedness and feelings of satisfaction with interpersonal relationships. The *Psychological Functioning* subscale focuses on key features of emotional/psychological functioning and asks about how the individual presently feels about their college experience.	

The most popular instrument which measures students’ adaptation to college multi-dimensionally is the Student Adaptation to College Questionnaire (SACQ) (Baker and Siryk, 1984). The instrument developed measures academic adjustment multifactorially: academic adjustment measures how well the student manages the demands of the college experience, social adjustment measures how well the student deals with interpersonal experiences at the academic environment, personal – emotional adjustment measures the extent that the student experiences general psychological distress and the somatic consequences of distress and institutional attachment measures the degree of institutional affiliation the student feels toward the university (Baker and Siryk, 1984).

There are few studies in Romania which measure academic adjustment; an instrument worthy of mention is the Academic Adjustment Questionnaire – AAQ (Clinciu and Cazan, 2014), its first version investigating three dimensions: Academic Neuroticism, Procrastination and Academic Dishonesty. However, the first attempt for creating an instrument to diagnose maladjustment led to the School Maladjustment Questionnaire (SMQ) (Clinciu, 2014). In academic context, maladjustment operationalized the reaction to the presumed stress due to the learning process through two correlative concepts: school neuroticism and rebel spirit. The initial instrument consisted of 67 dichotomous (true/false) items relevant to the students in secondary school and high school. Subsequent studies (Clinciu and Cazan, 2014) demonstrated the validity of the scale. Other research studies (Clinciu, 2019) revealed the necessity to create a new distinct instrument to investigate academic maladjustment at the university level as the experiences and demands students face considerably vary from those of secondary school and high school students. Furthermore, the previous studies have shown that an instrument that covers the multidimensional aspects of this phenomenon is more robust and can better capture the complex interplay between personal traits, academic prerequisite, academic adjustment and achievement.

Based on our experience in developing academic maladjustment questionnaires, we decided to create a broader and more detailed concept of this phenomenon, the new version of the instrument for university students encompassing an internal dimension (including anxiety, depression, and self-efficacy) as well as an external dimension (procrastination, academic dishonesty, and disruptive behavior). These dimensions will be combined to form a comprehensive and unified score.

## Materials and methods

Given the potential benefits of academic adjustment, the current study aimed to validate and evaluate a robust measure of overall academic maladjustment.

### Participants

The participants were 809 first-year students from various Romanian universities (M_*age*_ = 20.91, SD = 4.84), male (*N* = 165), and female (*N* = 634), non-binary (*N* = 7), not declared (*N* = 3), covering six fields of studies: Mathematics and Natural Sciences (*N* = 62), Sports Science and Physical Education (*N* = 23), Biological and Biomedical Sciences (*N* = 62), Engineering Sciences (*N* = 184), Social Sciences (*N* = 638), Humanities and Arts (*N* = 58), tuition-free students (*N* = 423), and tuition-fee paying students (*N* = 386).

### Procedure

The participants were recruited through email invitations, posts on the university website, the university social-media page and the internal communication app. The participation was voluntary, the survey invitation being available for all first-year university students. No incentives were offered for participation. The surveys were administered through LimeSurvey. The Ethics Committee in Social-Human Scientific Research at the Transilvania University of Brasov approved this study. This study was registered under this code: 31/21.09.2022. The main inclusion criterion was that the participants were students.

### Measures

Academic adjustment was measured with the Academic Adjustment Inventory (Clinciu and Cazan, 2014). The scale consists in 43 items, on a four-point Likert scale ranging from “not at all characteristic of me” to “extremely characteristic of me”. Higher values for this scale reflect a higher level of academic maladjustment. The initial structure of the scale consisted in three scales, with high Cronbach’s Alpha values: Academic Neuroticism (14 items, α = 0.92). Procrastination (11 items, α = 0.88), and Academic Dishonesty (19 items, α = 0.89). Cronbach’s Alpha for the entire scale was 0.93. Higher values reflect a higher level of academic maladjustment. The items are listed in Table 2.

**TABLE 2:** Factor loadings, reliability, and explained variances.

	Factor loadings						
	**F1**	**F2**	**F3**	**F4**	**F5**	**F6**	**F7**
24 I feel attracted by all sorts of small activities, which distract my attention from my school obligations.	0.835						
23 When I am at home, I have the habit of procrastinating starting doing my homework as long as I can.	0.765						
09 I often sit at my working desk reluctantly and vacantly.	0.748						
01 I enter a time crisis very often and that is why I frequently get with my homework being undone.	0.742						
12 I cannot organize either my time or my school activity.	0.665						
07 When we are unexpectedly given a current examination, I am almost sure that I shall not manage.	0.603		0.300				
02 I cannot follow the lectures in the classroom too long because my thoughts fly away	0.575						
*03 I often feel overwhelmed with the multitude of academic demands.*	*0.496*					*0.391*	
*08 Exams or current tests that are announced in advance put me under a deep state of pressure.*	*0.427*						
*04 There are subjects which make me feel incapable and helpless.*	*0.381*						
31 I don’t see any problem in letting someone else solve my course or seminar assignments.		0.856					
28 I have copied the assignment requested for a seminar or exam from a classmate before.		0.812					
32 I would be willing to let someone better prepared take an exam on my behalf.		0.747				0.373	
35 If I were in a time crisis, I would not hesitate to buy a pre-made thesis or dissertation.		0.633					
29 I have no problem with giving information to someone or allowing them to copy during an exam.		0.562					
27 I sometimes “draw inspiration” from my colleagues to solve homework or academic tasks.		0.533					
39 I wouldn’t have any problem “peeking” at my colleagues’ papers to better respond to the requests of an important exam.		0.502		0.378			
*33 I believe that some subjects are so difficult that it’s worth cheating to perform well in exams.*		*0.460*		*0.329*			
06 I hardly ever have the courage to solve a task in front of my colleagues, even if I know that I could do this thing correctly.			0.917				
15 When I am requested to answer at seminars, I become pale, I stammer, or I cannot easily find my ideas.			0.879				
16 I have a lump in my throat very easily when I have to answer at seminars.			0.859				
05 When I am requested to answer, I am caught by a state of deep panic and anxiety.			0.839				
41 In such a competitive world as today’s, I believe you have to be capable of anything to achieve good academic results.				0.851			
26 I believe that to achieve excellent academic results, “the end justifies the means.”				0.793			
42 I believe that used cleverly, lying can get you out of many college-related predicaments.				0.742			
43 The internet provides plenty of easy ways to get out of trouble at college, which I successfully use.				0.661			
40 I would be willing to commit some small dishonest acts (copying, whispering) to maintain my scholarship.		0.303		0.627			
10 I am exaggeratedly sensitive to criticism.					0.901		
11 Remarks and criticism (even the very small ones) deeply upset me and make me very angry.					0.850		
13 I cry very quickly out of nothing.					0.640		
*4 Sometimes I feel overwhelmed with states of sadness without any concrete reason.*					*0.330*	*0.320*	
18 I have an agitated and poor-quality sleep.						0.799	
19 In the morning I hardly wake up and I seem to be more tired than when I went to bed.						0.618	
20 Sometimes I am so fed up with everything.	0.345					0.582	
17 I have stomach pains, I hardly breathe or my heart beats insanely because of emotions.			0.481			0.533	
21 When I go to the faculty, I am late many times.							0.815
22 I have the tendency to spare time when I wake up and dress before going to the faculty.	0.465						0.539
30 I have invoked false excuses for the delay in completing a course or seminar assignment.							0.412
*37 If I saw someone cheating during an exam, I don’t think I could report it to the supervisor.*							
*34 I would have a guilty conscience if I used the same assignment to solve multiple seminar tasks.*							
*36 I believe that “inflating” the reference list with titles you haven’t read is a minor issue.*						*−0.317*	
*25 I know how to “win over” a teacher to gain personal advantages.*				*0.390*			
*38 A hyper-demanding teacher is worth being cheated on more than a permissive and ‘nice‘ one.*							
N of items	7	7	4	5	3	4	3
Eigenvalues	12.137	5.069	2.301	1.538	1.334	1.283	1.171
% of explained variance	28.225	11.788	5.350	3.576	3.102	2.983	2.724
Cronbach’s α	0.870	0.833	0.893	0.826	0.791	0.779	0.653

Rotation method: Promax with Kaiser normalization. Grey colored items were dropped.

Dropout intention was measured through a five-item scale developed for this research (i.e., *Sometimes, I think there are other professional fields that might suit me better than the one I am currently studying; I am considering giving up on this university; I intend to drop out of this university in the near future*), the items being measured on five-point Likert scale (ranging from strongly disagree to strongly agree), Cronbach’s Alpha being high, 0.82, high scores indicating a higher intention to dropout.

Two personality traits - neuroticism and conscientiousness - were measured using the IPIP scales (Iliescu et al., 2015). Each scale included 20 items on a five-point Likert scale, ranging from *not at all characteristic of me* to *extremely characteristic of me*. Alpha Cronbach’s values were high, 0.84 for the Conscientiousness scale (Example of items: *I have frequent mood swings; I am not easily bothered by things)* and 0.85, for the Neuroticism scale (Example of items: I follow a schedule; *I make a mess of things*).

A factual questionnaire was also used to collect data about the educational background: profile of the high school, baccalaureate mean grade (GPA), previous degrees or diplomas, the elapsed time from high school graduation, range of admission to the study program applied (first choice, second choice, etc.), and previous university enrolments.

### Data analysis

The questionnaires were administered during November – December 2022. Construct validity was estimated through Exploratory and confirmatory factor analyses. The convenience sample was randomly split into exploratory (*N* = 401) and confirmatory (*N* = 408) samples, the two halves not differing on gender [χ*^2^*(3) = 1.710, *p* = 0.635], type of enrolment tuition-free and tuition-paying students) [χ*^2^*(1) = 0.002, *p* = 0.963], or previous academic achievement [*t*(807) = 0.746, *p* = 0.456]. Exploratory factor analysis was computed using IBM SPSS 23.0, Promax with Kaiser Normalization being computed, and Confirmatory factor analysis was computed with IBM AMOS 23.0. Predictive validity was tested with linear regression, the dropout intention being the criterion. The Kaiser–Meyer–Olkin (*KMO* = 0.923) and Bartlett’s test of sphericity (χ*2* = 8817.738, *p* < 0.001) indicated that the data were suitable for factor analysis. For the CFA, parameters were computed through the maximum likelihood estimation method.

Multivariate normality of the data was determined by reviewing the absolute ranges for skewness and kurtosis, the values being less than 2.0, which indicates that the data is relatively normally distributed. The Mahalanobis distance was also checked, individual cases were analyzed (*p* values less than 1, showing the probability that the largest squared distance of any observation would exceed the Mahalanobis distance computed) but upon closer inspection, proved to be valid data points and, therefore, were retained in the data set.

Several fit indices: chi-square, CFI (Comparative Fit Index), TLI (Tucker–Lewis index), AIC (Akaike information criterion), RMSEA (Root Mean Square Error of Approximation) were used to evaluate model fit (Wang and Wang, 2019). Factor loadings, item-level statistics, and internal consistency were computed to investigate the academic adjustment factor structure. Invariance across gender groups was also computed, configural- (similar factor structures), metric- (similar factor loadings), and scalar (similar intercepts) models were compared. Invariance was determined through a non-significant difference in chi-square, CFI (1 < 0.01). To further investigate the predictive validity of the scale, correlations with grade point average and dropout intention were computed.

## Results

### Construct validity

#### Exploratory factor analysis (EFA)

Based on the eigenvalues, Kaiser’s rule, and the scree plot, a solution with seven to nine factors was assumed. The nine-factor solution covered 64.837% of the total variance. Although all the nine factors have eigenvalues higher than 1, the eighth and ninth factors included only 3, respectively, 2 items, with divergent meanings. Therefore, we decided to keep a seven-factor solution covering 57.748% of the variance (Table 2). A total of 10 items were deleted, given their low loadings or multiple loadings on different factors: the remaining 33 items lead to dimensions with high reliability, the Cronbach’s Alpha values being higher than 77, excepting the last dimension.

The seven factors were labeled as follows: F1 Procrastination, F2 Dishonesty – unethical behavior, F3 Test anxiety, F4 Machiavellian attitude, F5 Neuroticism, F6 Somatization, F7 Disengagement. There are two affective dimensions (neuroticism and test anxiety), a somatic dimension (somatization), four behavioral dimensions (procrastination, dishonesty, machiavellian attitude, and disengagement). However, the seventh factor has a low number of items, one of them having also a low loading (0.412) and a mixt structure (being saturated both in Factor 1 and 7). Therefore, the factor structure with and without factor seven will be tested in the confirmatory phase.

#### Confirmatory factor analysis (CFA)

Based on the EFA results, we confirmed the structure of the questionnaire through CFA. The fully correlated factor structure with all the factors converging to a general one showed good fit for some indices [root mean square error of approximation (RMSEA)] but low for others (comparative fit index (Table 3). The six-factor model had a better fit than the seven-factor model as expected by the EFA. Item 30 included in Factor seven had lower loadings compared with the other two, 0.348 for item 30, while for item 21 and item 22 the loadings were 0.627, respectively, 0.882. Therefore, the six-factor model was considered more appropriate.

**TABLE 3:** The goodness-of-fit indices for the measurement model.

Model	χ^2^/df	CFI	TLI	AIC	RMSEA
M1 – Seven-factor model	3.885***	0.809	0.793	2107.845	0.084
M2- Six-factor model – Uncorrelated errors	3.799***	0.833	0.818	1707.940	0.080
M3 – Six-factor model – Correlated errors	3.370***	0.862	0.846	1529.690	0.076

CFI, comparative fit index; TLI, Tucker–Lewis index; AIC, Akaike information criterion; RMSEA, root mean square error of approximation.

***p < 0.001.

The factor loadings for M3 did not reveal values lower than 0.384 (Table 4).

**TABLE 4:** Standardized estimates for the Confirmatory factor analysis (CFA) six-factor model.

Items	Dimensions	Loadings
F1 Procrastination	Academic maladjustment total	0.904
F2 Dishonesty – unethical behavior		0.384
F3 Test anxiety		0.721
F4 Machiavellian attitudes		0.443
F5 Neuroticism		0.693
F6 Somatization		0.885
24 I feel attracted by all sorts of small activities, which distract my attention from my school obligations.	F1 Procrastination	0.687
23 When I am at home, I have the habit of procrastinating starting doing my homework as long as I can.		0.705
09 I often sit at my working desk reluctantly and vacantly.		0.787
01 I enter a time crisis very often and that is why I frequently get with my homework being undone.		0.624
12 I cannot organize either my time or my school activity.		0.745
07 When we are unexpectedly given a current examination, I am almost sure that I shall not manage.		0.639
02 I cannot follow the lectures in the classroom too long because my thoughts fly away		0.653
31 I don’t see any problem in letting someone else solve my course or seminar assignments.	F2 Dishonesty – unethical behavior	0.710
28 I have copied the assignment requested for a seminar or exam from a classmate before.		0.743
32 I would be willing to let someone better prepared take an exam on my behalf.		0.599
35 If I were in a time crisis, I would not hesitate to buy a pre-made thesis or dissertation.		0.541
29 I have no problem with giving information to someone or allowing them to copy during an exam.		0.603
27 I sometimes “draw inspiration” from my colleagues to solve homework or academic tasks.		0.708
39 I wouldn’t have any problem “peeking” at my colleagues’ papers to better respond to the requests of an important exam.		0.782
05 When I am requested to answer, I am caught by a state of deep panic and anxiety.	F3 Test anxiety	0.781
16 I have a lump in my throat very easily when I have to answer at seminars.		0.913
15 When I am requested to answer at seminars, I become pale, I stammer, or I cannot easily find my ideas.		0.907
06 I hardly ever have the courage to solve a task in front of my colleagues, even if I know that I could do this thing correctly.		0.688
43 The internet provides plenty of easy ways to get out of trouble at college, which I successfully use.	F4 Machiavellian attitudes	0.553
42 I believe that used cleverly, lying can get you out of many college-related predicaments.		0.792
26 I believe that to achieve excellent academic results, “the end justifies the means.”		0.615
41 In such a competitive world as today’s, I believe you have to be capable of anything to achieve good academic results.		0.766
40 I would be willing to commit some small dishonest acts (copying, whispering) to maintain my scholarship.		0.731
13 I cry very quickly out of nothing.	F5 Neuroticism	0.628
11 Remarks and criticism (even the very small ones) deeply upset me and make me very angry.		0.865
10 I am exaggeratedly sensitive to criticism.		0.898
18 I have an agitated and poor-quality sleep.	F6 Somatization	0.636
19 In the morning I hardly wake up and I seem to be more tired than when I went to bed.		0.705
20 Sometimes I am so fed up with everything.		0.820
17 I have stomach pains, I hardly breathe or my heart beats insanely because of emotions.		0.665

Standardized estimates, *p* values <0.001 for all the items.

#### Results for invariance tests by student gender

Measurement invariance across genders (males: 166 vs. females: 639) was computed for the second-order CFA model, and the results were reported in Table 5. The results showed that all invariance models acceptably fitted the data. After that, the χ2 difference test suggested significant differences between the configural invariance model, the metric invariance model, and the scalar invariance model. Thus, we provided evidence for the configural invariance, meaning that the configuration of the indicators to their factors is the same across groups. However, model fit values did not decrease across the three invariance models. The significant differences between the configural and metric models could suggest that factor loadings are not equal across gender groups, however, the results should be interpreted cautiously, given the low number of male participants compared to female participants.

**TABLE 5:** Measurement invariance across genders.

Model	χ^2^	df	CFI	RMSEA	RSMEA CI_95_	TLI	Delta χ^2^
Configural invariance	2303.108	782	0.877	0.070	0.070−0.073	0.863	−
Metric invariance	2411.951	811	0.870	0.070	0.067−0.073	0.861	108.843***
Scalar invariance	2570.158	840	0.860	0.072	0.068−0.075	0.855	166.207***

CFI, comparative fit index; TLI, Tucker–Lewis index; RMSEA, Root Mean Square Error of Approximation.

***p < 0.001.

The correlations between the factors were positive and significant (between 0.16 and 0.69). The correlations of the scales with the total score were significant (between 0.63 and 0.84, *p* < 0.001), showing that the dimensions could be facets of the same construct (Table 6).

**TABLE 6:** Pearson correlations between academic maladjustment and its predictors.

	1	2	3	4	5	6	7	8	9	10
1 Procrastination	1									
2 Dishonesty – unethical behavior	0.406***	1								
3 Test anxiety	0.550***	0.151***	1							
4 Machiavellic attitudes	0.376***	0.699***	0.166***	1						
5 Neuroticism	0.467***	0.168***	0.563***	0.209***	1					
6 Somatization	0.628***	0.248***	0.575***	0.243***	0.532***	1				
7 Maladjustment total	0.845***	0.641***	0.697***	0.632***	0.651***	0.757***	1			
8 Dropout intention	0.412***	0.231***	0.255***	0.192***	0.200***	0.326***	0.394***	1		
9 GPA	0.056	−0.096**	−0.023	−0.079*	0.014	−0.011	−0.027	0.081*	1	
10 Neuroticism	0.522***	0.111**	0.553***	0.153***	0.532***	0.616***	0.581***	0.387***	−0.010	1
11 Conscientiousness	−0.264***	−0.306***	−0.404***	−0.264***	−0.330***	−0.445***	−0.602***	−0.410***	−0.078*	−0.512***

*N* = 805, ***Correlation is significant at the 0.001 level (2-tailed).

**Correlation is significant at the 0.001 level (2-tailed).

*Correlation is significant at the 0.05 level (2-tailed).

Previous performance (the admission grade) correlated weakly with the maladjustment. On the other hand, the two personality traits (neuroticism and conscientiousness) correlated significantly with maladjustment, the correlations being positive for neuroticism and positive for conscientiousness.

To test the concurrent validity of the scale, we used the dropout intention as criterion. All the dimensions and the total score of the maladjustment scale correlated positively and significantly with the dropout intention, procrastination and the total score being the strongest predictors. A simple regression analysis, using the overall maladjustment score as predictor led to a 15.5% of explained variance for the dropout intention, the regression model being statistically significant, F(1,804) = 147.68, *p* < 0.001. The academic maladjustment is a significant predictor (*B* = 0.562, *t* = 12.153, *p* < 0.001). Given the relatively high correlations between some of the maladjustment dimensions, we did not compute a multiple regression analysis (VIF values ranged between 1−2.08, showing that the variables are moderately correlated, which could raise concerns about the multicollinearity).

Gender differences were also computed, the independent *t* tests showing that men had higher levels for Dishonesty – unethical behaviors and Machavellic attitudes, while women had higher levels of Test anxiety, Somatization and Neuroticism. There were no significant differences for Procrastination and the overall score (Table 7).

**TABLE 7:** Gender differences for the maladjustment dimensions.

Dimensions		M	SD	t	df	sig	D Cohen
F1 Procrastination	Female	1.999	0.696	−1.955	794	0.051	0.088
	Male	2.118	0.703				
F2 Dishonesty – unethical behavior	Female	1.408	0.458	−6.008	206.490	<0.001	0.094
	Male	1.738	0.665				
F3 Test anxiety	Female	2.030	0.850	4.419	794	<0.001	0.090
	Male	1.706	0.794				
F4 Machiavelic attitudes	Female	1.556	0.588	−5.328	217.316	<0.001	0.092
	Male	1.897	0.765				
F5 Neuroticism	Female	2.087	0.871	8.094	313.385	<0.001	0.094
	Male	1.568	0.694				
F6 Somatization	Female	2.159	0.847	2.961	793	0.003	0.089
	Male	1.942	0.797				
F total	Female	1.822	0.505	−0.836	794	0.403	0.088
	Male	1.859	0.511				

## Discussion and conclusions

The purpose of this paper was to analyze the psychometric properties of a scale measuring academic maladjustment. The results showed that a second-order factorial model of overall academic maladjustment, comprised of six first-order factors called Procrastination, Dishonesty – unethical behavior, Test anxiety, Machiavellic attitudes, Neuroticism, and Somatization, fitted the data best. Our results, therefore, support the conceptual definition of academic maladjustment as a multifaceted concept, representing a dynamic and multidimensional process that can be explained by the fact that students enter college with certain personal characteristics (personality traits, motivation, academic prerequisites, and study skills) which are influenced by their interaction with the new environment (academic requirements, peers, professors, institutional culture) to establish a fit between their own demands, values, and expectations, and those of university life. The results further support partial measurement invariance across genders. Moreover, academic adjustment was substantially different but related to academic dropout intention. Furthermore, academic maladjustment was negatively associated with conscientiousness and was positively related to neuroticism. The correlation with academic performance was weak.

### The academic maladjustment framework

Academic maladjustment remains a rather controversial issue, and even if it is defined in contrast to academic adjustment, the definitions reveal a multifaceted concept, representing a dynamic and multidimensional process, referring to a set of behavioral, social, and emotional difficulties that prevent students from adapting to the new learning environment, coping successfully with academic demands, achieving performance, and academic engagement (Jansen and van der Meer, 2012), in its extreme form, leading to academic dropout. Stressors present at the university, lack of social support, low self-regulation skills, low academic expectations, low fit between students’ personal and vocational interests and goals and faculty choice are both indicators of academic maladjustment but also significant predictors of dropout risk and intention (Casanova et al., 2021). Students who struggle to cope with stressors and fail to overcome the initial challenges of adaptation tend to show lower engagement in academic tasks and campus life; they may experience reduced levels of academic achievement and satisfaction, or higher levels of emotional exhaustion and anxiety when confronted with these difficulties (van Rooij et al., 2018). As can be observed, the majority of studies use the concept of academic adaptation, but its description and definition primarily highlight the negative aspects, such as high levels of stress, low social support, deficient autonomy and self-regulation, etc. Therefore, we consider it relevant to use the concept of academic maladjustment, which can better encompass the aspects mentioned earlier. Therefore, the proposed model of academic maladjustment includes negative affectivity dimensions (neuroticism and test anxiety), somatic symptoms (somatization), and negative or counterproductive behaviors (procrastination, dishonesty, machiavellian attitude). As previous studies showed, negative feelings and behaviors tend to reduce self-efficacy perceptions, increase academic dissatisfaction, and are related to dropout intention (Sinval et al., 2021; Stajkovic et al., 2018). Previous studies also emphasize negative emotionality as a possible dimension of maladjustment (Clinciu, 2013). The previous cited work defines adaptation by starting from its opposite, which is academic maladjustment. This involves internal reactions that are difficult to see or invisible on the surface (such as school-related anxiety and depression, issues with self-esteem, etc.) or external reactions that are visible on the outside, such as disruptive tendencies, rebellious spirit, school dropout, academic dishonesty, etc (Clinciu, 2013). Therefore, negative emotionality can be considered an important component of maladjustment.

A new aspect is represented by the introduction of academic dishonesty into the model of academic maladjustment, which has been less discussed in the literature. Even though the initial concerns of the authors regarding the integration of the academic dishonesty dimension into the structure of academic maladjustment date back a few years (Ives et al., 2017; Clinciu et al., 2021) the results of the current study support the fact that this dimension is strongly involved in explaining this concept. The cited studies showed that students who experience difficulties in adapting to the academic environment may be more inclined to engage in dishonest behaviors as a coping mechanism or due to a lack of self-efficacy in their academic pursuits. Academic dishonesty could be used as a coping mechanism for students who feel overwhelmed or inadequate in the face of academic challenges, and they may resort to cheating or plagiarism to avoid failure or meet perceived expectations; the desire to achieve associated with a high course load and external pressures produce conditions to engage in academic dishonest behaviors (Geddes, 2011). As a result, engaging in academic dishonesty can lead to negative consequences, such as academic penalties, loss of trust from teachers and peers, and damage to one’s academic reputation, which are also indicators of maladjustment. The structure of the new dimension and its weight in the final instrument was therefore tested and validated.

### Psychometric properties of the academic adjustment questionnaire

In the specialized literature in Romania, there are few concerns regarding the measurement of academic (in)adaptation, the most well-known being materialized in the creation of the Academic Adjustment Questionnaire – AAQ (Clinciu and Cazan, 2014). The authors’ initial approach was continued by adding a new dimension, academic dishonesty. Additionally, the authors chose to modify the dichotomous response scale by evaluating the items on a four-point Likert scale, which necessitated retesting the psychometric properties of the scale and analyzing its factorial structure on a more diverse sample of students. The results showed a reliable version of the instrument with a factorial structure that did not deviate significantly from the authors’ initial model (Clinciu, 2019). While the theoretical model referred to an internal dimension with three components, anxiety, depression, and self-efficacy and an external dimension with three components: procrastination, academic dishonesty, and disruptive behavior, the exploratory and confirmatory factor analysis revealed a unified score including six dimensions, procrastination, dishonesty – unethical behavior, test anxiety, machiavellian attitude, neuroticism, and somatization. The behavioral aspect focused on procrastination as expected, the dishonest behavior items grouped in two types of behaviors: unethical behaviors (letting someone else solve his/her assignments, copying the assignment from a classmate etc.) and machiavellian attitudes (adopting the attitude the end justifies the means, lying to get out of college-related predicaments etc.); while the first component refers to dishonest misconduct in the form of cheating on assignments, plagiarism, or unacceptable collaboration, the second components includes also moral values (accepting lying as acceptable behavior, accepting reprehensible behaviors or obtaining academic rewards), therefore the label of this dimension included a moral meaning to differentiate it from common dishonest behaviors. Previous studies confirmed a considerable overlap between machiavellism and dishonest academic behaviors (Barbaranelli et al., 2018; Esteves et al., 2021), confirming that students engage in academic misconduct, due to their proneness to disregard norms and rules for their own benefit. Therefore, the disruptive behavior component could be considered a form of machiavellian attitude.

Not surprisingly, procrastination seemed to be one of the best-represented factors of maladjustment, with the highest eigenvalue and the highest explained variance, convergent with previous studies which defined procrastination as a common phenomenon that disrupts academic responsibilities (Grunschel et al., 2013), academic procrastination represents an irrational and frequently detrimental form of delay, significantly linked to adverse effects on academic performance, health, or emotional well-being (Ziegler and Opdenakker, 2018).

Negative emotions as a dimension of academic maladjustment proved also to be a relevant component, operationalized as test anxiety and neuroticism. Studies have demonstrated a connection between emotions and academic achievement. Generally, positive emotions, like finding joy in learning, are associated with higher levels of achievement. Conversely, negative emotions, such as experiencing test anxiety, are linked to lower academic performance (Lichtenfeld et al., 2022). The academic environment brings challenges that students must overcome to acquire new skills. When students experience negative emotions, they often encounter difficulties with concentration, low self-esteem, and a lack of energy, they tend to face more issues with their college work and lack of motivation (Iglesias-Benavides et al., 2016). Therefore, higher levels of negative emotions as a dimension of academic maladjustment is often discussed in the literature. Our results revealed that both neuroticism and test anxiety have an important weight in the assessment of maladjustment, test anxiety being a stronger factor, as expected and highlighted also but recent studies showing a negative link between test anxiety and academic achievement (Steinmayr et al., 2016). The construct validity of these factors is also confirmed by the high negative and significant correlations with neuroticism as personality trait. On the other hand, conscientiousness was negatively related to maladjustment and its dimensions, test anxiety and somatization being more significant correlated. Previous studies confirmed that high neuroticism and conscientiousness are associated with high levels of cognitive test anxiety, students with low emotional stability being more prone to experiencing elevated levels of anxiety in evaluative situations. This susceptibility may be due to their heightened self-conscientiousness, tendency toward depression, and limited emotional regulation skills (von der Embse et al., 2018; Thomas and Cassady, 2019).

The crystallization resulting from the factorial analysis of a factor synthesizing somatic complaints is not coincidental, previous studies suggesting that somatic complaints are often indicators of school maladjustment (Otterpohl et al., 2017), somatic complaints being considered as a way of expressing psychological difficulties. Although physical complaints such as fatigue, abdominal pain, sleep disorders or headaches are more commonly reported during childhood, our study revealed that they could be encountered also in university students.

The low correlations between previous academic performances and maladjustment are not surprising, previous studies confirming these results (Stan et al., 2023). Our results confirm that besides academic achievement, personal factors are important indicators of adjustment, showing that personal resources management, emotional and behavioral strategies are components of adjustment. A future study will focus on the predictive value of academic maladjustment on future academic performances, given the fact that previous research showed that academic performance could serve as an objective measure of college students’ adaptation outcomes (Li et al., 2023).

To test the predictive validity of the instrument, academic dropout was used as a criterion, dropout intention being frequently used in predictive models of academic adjustment (Respondek et al., 2017; López-Angulo et al., 2023). Our study revealed a medium and positive correlation between overall maladjustment and academic dropout intention (r^2^ = 0.152); procrastination seemed to be the most relevant predictor of dropout intention, the correlation with dropout intention being higher than for the other maladjustment dimensions. Academic adjustment acts as a safeguard against dropping out, and it is crucial to acknowledge that most students enter college with the intention of completing their studies (Cădariu and Rad, 2023). Nevertheless, various factors can lead to dropout, such as opting to pursue education in another city, or deciding to focus on a vocational path instead, working while studying, therefore these variables could be used as moderators in future studies. Understanding these reasons is essential for devising effective retention strategies and supporting students throughout their academic journey. The predictive value of the AAQ for the dropout intention has important practical implications. While international and national strategies exist to enhance student retention, personalized follow-up and data collection from students who may not actively seek university support due to their unique characteristics are often lacking. As a solution, the AAQ could play a role in monitoring students from enrollment to graduation, enabling the implementation of effective, efficient, and timely interventions. This approach would identify deficient areas, allowing targeted actions and early detection to ultimately prevent dropouts (Donado et al., 2021).

## Limitations and future research directions

Despite the novelty of the current study, it is not without its limitations. First, the collected data were cross-sectional. Therefore, the scale’s stability over time is unknown. However, a future study will investigate this aspect, a second round of data being collected after the end of the first academic year. Second, all indicators relied upon self-report measures, which cannot entirely overcome the positive bias. Future research should aim to validate the scale against more objective indicators of academic well-being, learning engagement, and academic performance. Thirdly, the current study checked the measurement invariance of academic maladjustment across gender. However, academic maladjustment may also vary across age and field of study. Future researchers could examine the factorial equivalence of the Academic Maladjustment Scale across these factors. The measurement non-invariance for metric and scalar invariance suggests that the construct of academic maladjustment has a different structure or meaning to gender groups, however, the number of male participants in this study is low, the sample being unbalanced regarding gender, which can affect the results. Future studies will include a higher number of male participants and also a more balanced sample regarding the field of study in order to re-test the measurement equivalence of the instrument. Finally, the cross-cultural validity of the instrument should be investigated. In the current study, the empirical validation of the instrument was only conducted within the Romanian European context, therefore, future studies should attempt to validate the scale in other cultural contexts in order to provide more evidence as to its cross-cultural applicability.

## Conclusion

The development and validation of the Academic Maladjustment Questionnaire represents a significant Contribution To The Field of educational psychology. Firstly, the inclusion of behavioral (procrastination and unethical behavior) and moral aspects (machiavellian attitudes) is highly relevant as it offers a more comprehensive understanding of students’ difficulties and challenges in adapting to the academic demands of the university environment. Previous studies have often approached dishonest behavior as being associated with an individual tendency to overlook norms, but the use of the new instrument might shed more light on the complex relationship between academic demands and academic dishonesty among first-year students. This has practical implications as well, higher education institutions can use the instrument as a toolkit to assess the tendency to engage in procrastination and unethical behavior in direct response to challenges and demands of the academic environment. The deeper understanding and acknowledgment of these behaviors can serve in designing and implementing interventions to prevent or diminish procrastination and unethical behaviors among students and in promoting a culture of ethics within the university.

Secondly, the exploration of the six distinct dimensions of academic maladjustment, including procrastination, dishonesty, test anxiety, machiavellian attitudes, neuroticism, and somatization, reveals the multifaceted nature of this construct. This multifaceted approach can help researchers and university staff to better address the various aspects that can facilitate students’ successful adaptation to university life. By understanding both the behavioral and emotional dimensions of academic adjustment, universities can target better their interventions. By recognizing the specific aspects that students experience or struggle with, tutors, educational counselors and even teachers can personalize the support they offer to first-year students. For example, students who experience intense negative emotions in relation to evaluation may benefit more from targeted educational and psychological counseling, while students who confront more with somatization may also need medical support and long-term educational or psychological counseling.

Finally, the Academic Maladjustment Questionnaire has significant implications for educational practice as it can be used to identify students at-risk of dropout. As all the dimensions and the unified score correlated positively with dropout intentions, universities can early recognize students experiences academic maladjustment and address the issue in early interventions. By doing this, they can potentially reduce the high dropout rates in first-year students and can also enhance academic performance of those students by addressing their difficulties early on in the course of their studies. Procrastination was the strongest predictor of dropout intention, sustaining once more the need for targeted intervention on multiple facets of academic maladjustment.

In conclusion, our first attempt to conceptualize and measure academic maladjustment has shown promising results. Our results support the importance of measuring academic maladjustment as an indicator of dropout intention and academic success. The effectiveness of the newly developed instrument will be confirmed through its implementation in prevention, counseling, and academic stress management activities. Moreover, its use in transcultural studies will provide a more accurate representation of culturally specific constructs being measured. Preventing academic maladjustment could therefore be an important aim of alternative programs and strategies that researchers and practitioners could implement to sustain student efforts’ toward a successful transition to university and professional life.

## Data availability statement

The raw data supporting the conclusions of this article will be made available by the authors, without undue reservation.

## Ethics statement

The studies involving humans were approved by the Ethics Committee in Social-Human Scientific Research at the Transilvania University of Brasov. The studies were conducted in accordance with the local legislation and institutional requirements. The participants provided their written informed consent to participate in this study.

## Author contributions

A-MC: Conceptualization, Data curation, Formal analysis, Funding acquisition, Investigation, Methodology, Project administration, Writing – original draft, Writing – review and editing. MMS: Investigation, Methodology, Writing – original draft, Writing – review and editing, Conceptualization. AIC: Supervision, Validation, Writing – original draft, Writing – review and editing. CT: Investigation, Resources, Visualization, Writing – original draft, Writing – review and editing, Methodology. CIM: Data curation, Formal analysis, Methodology, Software, Writing – review and editing.
